# Ultra-broadband, wide angle absorber utilizing metal insulator multilayers stack with a multi-thickness metal surface texture

**DOI:** 10.1038/s41598-017-04964-3

**Published:** 2017-07-06

**Authors:** Amir Ghobadi, Sina Abedini Dereshgi, Hodjat Hajian, Berkay Bozok, Bayram Butun, Ekmel Ozbay

**Affiliations:** 1 NANOTAM-Nanotechnology Research Center, Bilkent University, 06800 Ankara, Turkey; 2 Department of Electrical and Electronics Engineering, Bilkent University, 06800 Ankara, Turkey; 3 Department of Physics, Bilkent University, 06800 Ankara, Turkey; 4 UNAM-Institute of Materials Science and Nanotechnology, Bilkent University, Ankara, Turkey

## Abstract

In this paper, we propose a facile route to fabricate a metal insulator multilayer stack to obtain ultra-broadband, wide angle behavior from the structure. The absorber, which covers near infrared (NIR) and visible (Vis) ranges, consists of a metal-insulator-metal-insulator (MIMI) multilayer where the middle metal layer has a variant thickness. It is found that this non-uniform thickness of the metal provides us with an absorption that is much broader compared to planar architecture. In the non-uniform case, each thickness is responsible for a specific wavelength range where the overall absorption is the superposition of these resonant responses and consequently a broad, perfect light absorption is attained. We first numerically examine the impact of different geometries on the overall light absorption property of the multilayer design. Afterward, we fabricate the designs and characterize them to experimentally verify our numerical findings. Characterizations show a good agreement with numerical results where the optimum absorption bandwidth for planar design is found to be 620 nm (380 nm–1000 nm) and it is significantly boosted to an amount of 1060 nm (350 nm–1410 nm) for multi-thickness case.

## Introduction

Electromagnetic wave absorbers are a field of study that has gained great interest in recent years. A variety of applications, including sensing^1–5^ and spectroscopy^6, 7^, photovoltaic and thermal photovoltaic^8–11^, and photo detection^12–15^, are the subject of this area. Owing to their exceptional optical properties, sub-wavelength metamaterials have been dominated among other absorbing architectures^16^. These structures are utilized in different frequency ranges spanning from microwave to optical regime. Using nano patterned patch in a meal-insulator-metal (MIM) configuration was one of the most frequently employed ideas to obtain perfect light absorption^17^. In general, inherent light absorption in these MIM designs are due to magnetic and electric dipoles occurring at certain narrow wavelength ranges^2, 18–32^. In this configuration, the light reflects back from the bottom metal layer and in a specific wavelength range the reflected light phase is destructive with that of incoming light, and consequently overall light reflection approaches zero. However, as mentioned earlier, these nano resonant structures have narrow band light absorption that is a limiting factor in several applications where a broad light absorption is a must. To overcome this limit, several different efforts have been devoted to fabricate a design that provides light absorption in a broad frequency range. The use of multi-shaped (or multi-sized) resonant structures, where the superposition of several adjacent resonances will make an overall flat and broad absorption, is one of the common methods^11, 33–42^. Another approach is to use ultra-sharp features as the top layer to ensure light confinement in the design^41^. Furthermore, employing tapered or pyramid architecture for gradual matching between air and device impedances is another repeatedly accomplished method to obtain perfect broadband light absorption^11, 43–50^. However, most designs suffer from fabrication complexity and large scale production incompatibility due to the existence of sub-wavelength nano resonant units that can be made by Electron-beam lithography (EBL).

The use of ultrathin lossy metals as the top layer in a MIM configuration was a solution to tackle with fabrication complexity. In a previous study, it was demonstrated that the use of a ~3 nm Chromium (Cr) top layer in Cr-SiO_2_-Cr stack can provide an absorption above 0.95 from 450 nm to 850 nm^51^. Recently, it was theoretically demonstrated that the use of metal-insulator (MI) multilayer with proper layer thicknesses can provide light absorption in a broad wavelength range where the absorption edge can be extended toward longer wavelengths by adding the number of pairs up^52^. This design configuration does not need any nano patterning that ensures its production feasibility. After this proposed design, several experimental efforts are exploited to fabricate a perfect ultra-broadband absorber based on an MI multilayer. Different types of metal and insulators are employed to attain ultra-broadband absorption from these multilayer stacks. W-Al_2_O_3_^53^, Cr-SiO_2_^54^, W-SiO_2_^54^, and Ni-SiO_2_^55^ are some examples of these MI pairs to obtain perfect broadband absorption. Deposition of metal layers is commonly employed using physical vapor deposition (PVD) methods, such as thermal evaporation and sputtering. However, considering the ultrathin thickness of the metal layer, these methods are not as repeatable as chemical vapor deposition (CVD) methods wherein the use of a gas precursor will ensure a pin hole free ultra-smooth layer that is also large scale fabrication compatible. Among the different CVD methods, atomic layer deposition (ALD) is an excellent choice for the deposition of highly smooth metal layers. Atomic layer deposition is a self-limiting growth process that offers uniform and conformal coating of non-line-of-sight surfaces including high aspect ratio features. This method ensures the uniform and conformal coating of pinhole-free, nanometer-thick layers over the entire substrate surface, which is imperative for improving material/device performance.

In a common design, where both metal and insulator layers are simple planar structures that are stacked together, the overall response of the layer depends on metal and insulator layer thicknesses. This dependence is even sharper for metal coating. The findings illustrate that thin layers have better absorption in longer wavelengths and thick ones have stronger absorption in short λs. Therefore, a wise approach is to exploit both characteristics in the same design where strong light absorption and lager bandwidth can be attained. It should be considered that the fabrication route of this stack needs to be large scale compatible where the EBL-free characteristic of the design is not diminished.

Herein, we propose a simple facile fabrication route to obtain an ultra-broadband wide-angle light absorber based on a multi-thick textured metal layer. Unlike previously reported designs that use a simple planar multilayer structure, the proposed architecture is a metal-insulator-metal-insulator (MIMI) stack where the middle metal layer is a thickness variant layer to provide broad light absorption. First, the growth of a bottom metal-insulator-metal multilayer is carried out by ALD. Afterward, the multilayer stack is turned into a nanodot/nanohole morphology utilizing a simple annealing step. Finally, the sample is put back into an ALD chamber and upper metal and top insulator layers are grown on top of this design. It is in this way that a thickness variant metal layer can be obtained. Our findings prove that the efficient choice of metal layer thicknesses can offer a bandwidth that is much larger compared to that of a planar design. This methodology can be employed in other MI multilayer structures where a much broader response can be obtained without changing the overall thickness of the stack. The main potential applications of the proposed design can be comprised of thermal photovoltaic and photo detection (if properly designed). Particularly, obtaining ultra-broadband perfect absorption is of great importance for thermal photovoltaic application. Moreover, considering the high operating temperature of the device, a refractory metal with high melting temperature is required to have stability and durability against corrosion and oxidation. Specifically, Pt as our choice of metal, has a high melting temperature and non-oxidizing nature which makes it an excellent option for thermal photovoltaic.

## Methods

### Synthesis of Metal-insulator Pair by Atomic Layer Deposition

Briefly, first, quartz substrate is cleaned in a sonication bath with acetone, ethanol, and de-ionized (DI) water each for 15 min and then dried by N_2_ flow. Then, a Pt- Al_2_O_3_ multilayer is grown on this substrate using the ALD method. Both Pt and Al_2_O_3_ depositions are carried out at 250 °C in an ALD reactor (Cambridge Nanotech Savannah S100) employing Al(CH_3_)_3_ and MeCpPtMe_3_ solution as deposition precursors, respectively. For the case of a Pt layer, the pulse and purge times are chosen to be 0.1 s and 15 s while for Al_2_O_3_, these amounts are 0.015 s and 10 s. As an oxygen source, water and ozone are utilized for Al_2_O_3_ and Pt, respectively. In the multilayer design, the bottom Pt layer thickness is fixed at 50 nm. The other three layers thicknesses are adjusted by controlling the number of deposition cycles. The growth rate for Pt and Al_2_O_3_ layers is estimated to be 0.93 Å per cycle and 1.08 Å per cycle, respectively.

### Nanohole and Nanoisland Formation Using the Dewetting Process

To form nano structures from a thin metal layer, we utilized furnace based annealing. The setup is made of a quartz tube integrated into a vacuum pump. The annealing is performed in a vacuum environment (with a base pressure of ~10^−3^) to prevent oxidation of the layers. In this method, the sample is put in the tube furnace and the temperature is raised up to 800 °C at a rate of 30 °C/min. Then, it is kept at this temperature for our desired duration for dewetting treatment and after that duration the furnace is opened to room temperature for fast cooling. The fast cooling step is critical to form nanoislands. Because under a slow cooling process, the melted part of the metal can rearrange to its layer morphology and formation of nanoislands would be prevented.

### Characterization

Scanning electron microscope (SEM, FEI – Quanta 200 FEG) operated at 10 kV is used for top and cross sectional imaging. For the optical characterization of the stacks, the normal reflection measurements for the wavelength range of 600 nm to 2000 nm were carried out using Fourier Transform infrared spectroscopy (FTIR, Bruker) and for the remaining part of the visible spectrum, we used a homemade reflection measurement setup in which a Halogen illuminator connected to a microscope and directed perpendicular to a sample and the reflected light from the microscope was fed to a Newport OSM2 spectrometer and the data was collected by interfacing the spectrometer with PC. The angle resolved reflection characterization was carried out using J.A. Woollam Co. Inc. VASE ellipsometer for different angles of incidence and polarizations.

## Results and Discussion

**Figure 1:**
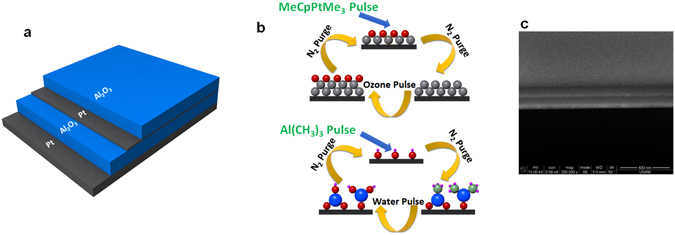
Schematic illustration of (**a**) proposed Pt-Al_2_O_3_ MIMI structure, (**b**) the corresponding single ALD cycle to deposit Alumina and Platinum layers, and (**c**) cross sectional SEM image of fabricated planar multilayer stack.

Figure 1a shows a schematic description of the fabricated Pt-Al_2_O_3_ stack. The bottom Pt layer is chosen to be 50 nm thick to act as a mirror, where the incoming light is entirely reflected back to the cavity, in the operation frequency range. On top of this thick Pt layer, a symmetric Al_2_O_3_- Pt- Al_2_O_3_ multi-layer is coated where the thickness of the insulator (D_I_) is taken to be identical for both layers and a thin Pt layer with a thickness of D_M_ is sandwiched in between. To be able to obtain metal full coverage in such a thin layer, we have utilized ALD. ALD is a self-limiting growth process where a gaseous precursor is utilized to expose the substrate surface and it offers uniform and conformal coating of a pinhole-free, angstrom-thick layer over the entire substrate surface. However, typical physical vapor deposition (PVD) techniques have less control on film morphology and obtaining a continuous layer can be achieved usually in thicker layers (>5 nm). On the other side, the use of ALD process for both metal and insulator layers gives us this opportunity to grow multilayer stack in a single process. This in turn offers a highly repeatable, high throughput growth in a full wafer scale. It should be noted that although ALD process is a slow process, the use of gaseous precursor provides an opportunity to coat samples in a large scale. An illustrative image is also shown in Fig. 1b to explain the preparation route of Al_2_O_3_ and Pt layers. In brief, in every cycle, the first oxygen source (water for Al and Ozone for Pt) is pulsed into the system and by this way OH chemical groups will be chemisorbed onto the surface. In the second half cycle, material precursor (Al(CH_3_)_3_ for Al and MeCpPtMe_3_ for Pt) is pulsed into the chamber to form Al_2_O_3_ and Pt layers. The details of the ALD process are provided in the methods section. A cross sectional SEM image of the fabricated stack is illustrated in Fig. 1c that shows the successful growth of a multilayer stack.

In the first step to study this multilayer, the impact of insulator and metal thicknesses in the overall absorption of the system is scrutinized using numerical calculations by employing a commercial finite-difference time-domain (FDTD) software package (Lumerical FDTD Solutions)^56^. In the numerical simulations, the propagation direction of incident light is chosen to be perpendicular to the x–y plane. Periodic boundary conditions are also employed in the x and y directions, while boundaries in the z direction are adopted as a perfectly matched layer (PML). A broadband plane wave with the polarization of the electric field along the x axis being utilized to stimulate the unit cell and reflected (R) and transmitted (T) lights are calculated by two monitors on the two sides of the multilayer stack. Therefore, absorption (A) is calculated by using the equation of *A* = 1 − *R*−*T*. Considering the fact that the bottom Pt layer thickness is twofold thicker than that of light skin depth at our operation frequencies, we can suppose transmission T to be zero (this has been verified during our simulations). Consequently, the absorption can be calculated by the following simplified equation of *A* = 1 − *R*.

**Figure 2:**
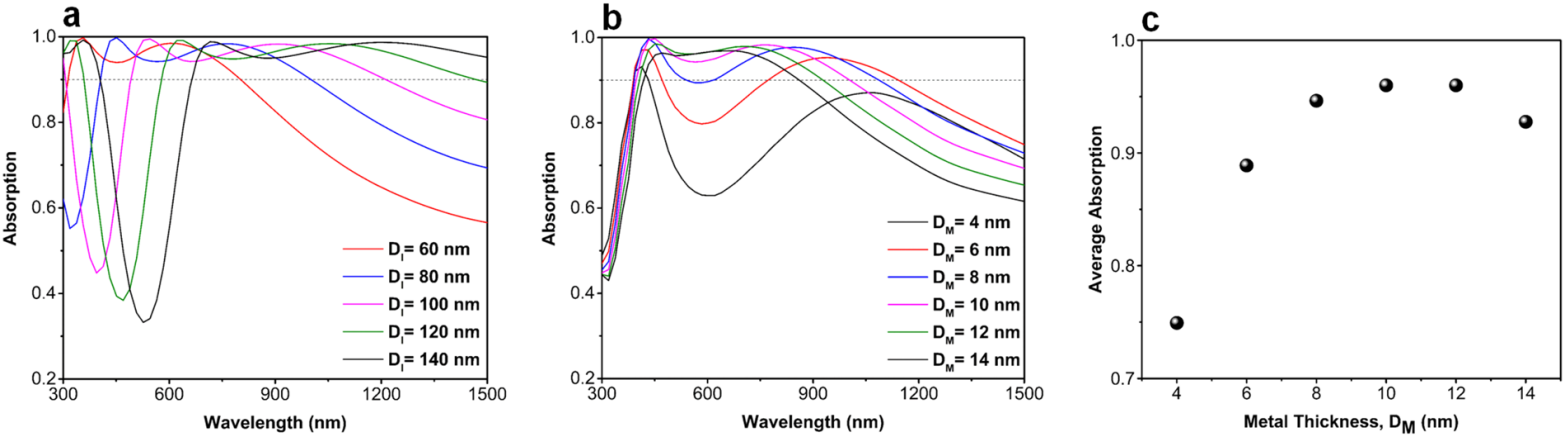
Absorption spectra of the fabricated MIMI stack for different (**a**) insulator layer thicknesses (D_I_s) and (**b**) metal layer thicknesses (D_M_s). Part (**c**) depicts the average absorption values for different MIMI configurations.

The simulated absorption spectra of the proposed broadband MIMI absorber as the function of insulator layer thickness (D_I_) are plotted in Fig. 2(a). It should be mentioned that in these spectra the metal layer thickness D_M_ is fixed at 10 nm. As it can be clearly seen in this figure, the absorption experiences a sharp reduction in lower wavelengths where the position of this lower edge is red-shifted for larger D_I_ values. Keeping the perfect absorption threshold as 0.9, structures with thicker layers have a longer absorption edge at larger wavelengths but loose their absorption edge in lower ones. In order to cover whole visible frequency range, the insulator thickness D_I_ is chosen to be 80 nm. Keeping this value as the insulator layer thickness, this time, we sweep D_M_ values from 4 nm to 14 nm. As plotted in Fig. 2b, the absorption capability of the thin metal layers is better in longer wavelengths while a perfect flat absorption in shorter wavelengths can be achieved in thicker metal ones. To have a better qualitative comparison, the average absorptions, in a wavelength range of 400 nm–1000 nm for different D_M_ values, are calculated and illustrated in Fig. 2c. An absorption above 0.95 is obtained for D_M_ values of 10 nm and 12 nm. Considering the bandwidth as the second figure of merit, we found that the best results are attained from 80-10-80 case (throughout the paper ‘insulator layer thickness (nm) - metal layer thickness (nm) - insulator layer thickness (nm)’ notation will be used to address different configurations). The contour plots for different metal and insulator layer thicknesses are also in the supplementary information (Fig. S1).

To evaluate these findings with the theoretical calculations, a modeling methodology is utilized to explain these results. As theoretically illustrated in a previously reported study^54^, a broadband absorption response can only be satisfied if the impedance matching condition is provided for the MIMI structure throughout a broad frequency range. This means that the normalized input impedance Z_in_ of the perfect absorber should be around unity (*Z*_*in*_ = 1Ω) in the frequency range of interest. To meet this condition, considering the bottom metal layer as a perfect reflector, we need an ideal metal in which the real and imaginary parts of its permittivity are described as:

1 $${\varepsilon }_{ideal}^{^{\prime} }(\omega )=\frac{-{\varepsilon }_{A{l}_{2}{O}_{3}}C}{{n}_{m}^{^{\prime\prime} }\omega {d}_{Pt}},{\varepsilon }_{ideal}^{^{\prime\prime} }(\omega )={\varepsilon }_{A{l}_{2}{O}_{3}}\frac{({n}_{m}^{^{\prime\prime} }C-{\varepsilon }_{A{l}_{2}{O}_{3}}\omega {d}_{Pt})}{{n}_{m}^{^{\prime\prime} }\omega {d}_{Pt}}$$

**Figure 3:**
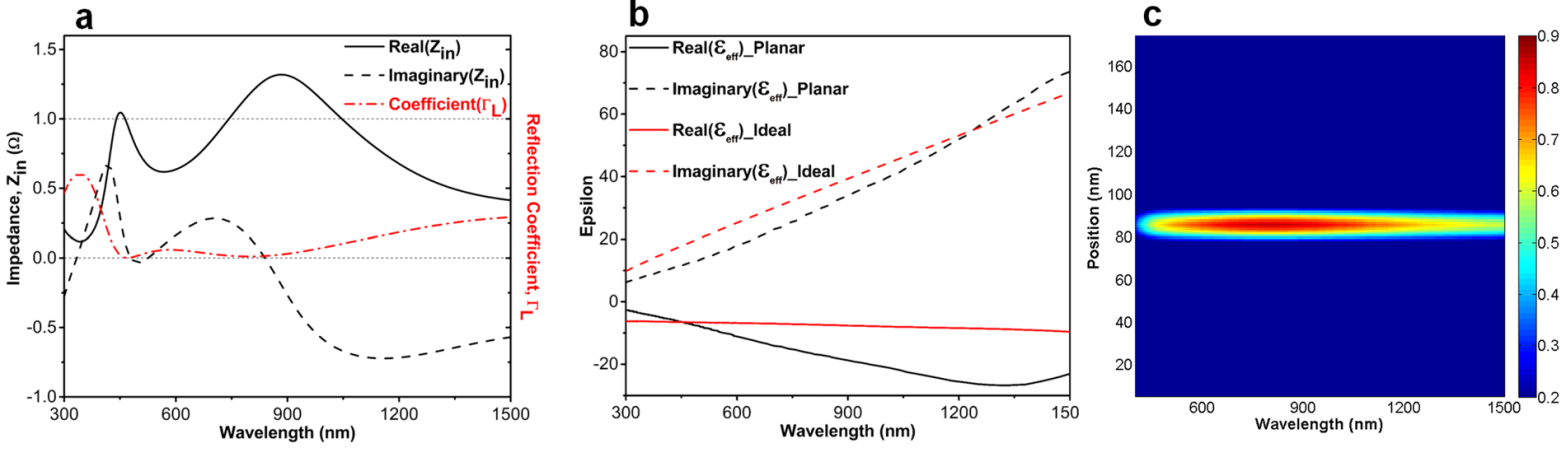
The extracted values for (**a**) input impedance and reflection coefficient of the MIMI structure, (**b**) real and imaginary part of permittivity for ideal and fabricated planar cases and (**c**) the absorption distribution over the MIM stack.

Here $\,{n}_{m}^{^{\prime\prime} }$ is the imaginary part of the refractive index of the metallic reflector, and C stands for the speed of light in free space. Thus, a metal whose permittivity value is matched to these values can guarantee a broad absorption response. To see how well it matches for our MIMI case, we extracted input impedance values (both real and imaginary parts) of the absorber using the well-known effective parameter extraction method^57^ (Fig. 3a). In order to have a better comparison, the absolute value of reflection coefficient (${{\rm{\Gamma }}}_{L}=\frac{{Z}_{in}-1}{{Z}_{in}+1}$) of the structure is also determined and depicted in Fig. 3a. As it is clearly illustrated, the reflection coefficient has its minima at λ = 470 nm and λ = 800 nm that are the places that the real part of *Z*_*in*_ is about unity. This, in turn, reveals how the impedance matched conditions can be attributed to the zero reflection points. This impedance matched condition suggests that the permittivity of the Pt fairly meets that of an ideal metal which is described by Eq. 1. This agreement between the epsilon values of ideal metal and that of Pt has been proved in Fig. 3b. Therefore, when light is cast on the multilayer, due to good impedance matching what Pt provides, the light does not reflect back and it is absorbed in the metal layer as plotted in Fig. 3c.

**Figure 4:**
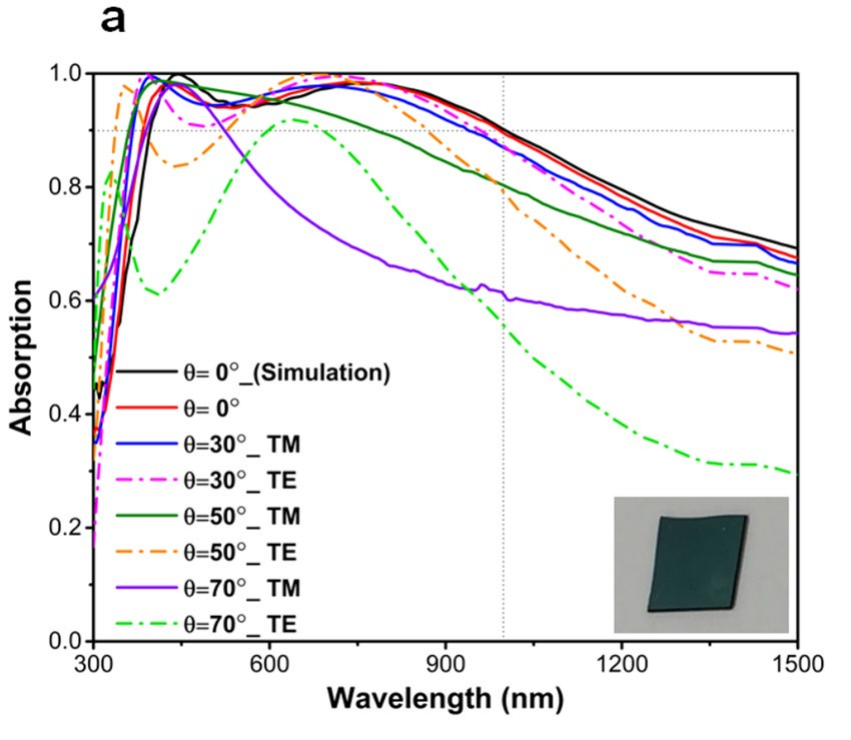
The simulation and measurement spectra for normal and oblique angle incident light with different polarizations of TE and TM.

To experimentally validate our numerical findings, we have fabricated the optimal configuration of 80-10-80 using a single ALD step. The absorption spectra for normal incident light and lights with different angles of incidence and polarizations (transverse magnetic (TM) and transverse electric (TE)) are depicted in Fig. 4. It is shown that the simulation result for normal incident light is in excellent agreement with that of obtained by measurement, providing a bandwidth of about 620 nm, which extends from 380 nm to 1000 nm. The angular dependence of the absorption spectra for TE and TM polarizations in different incident angles of *θ* = 30°, 50°, 70° are also drawn in this figure. It is seen from Fig. 4 that for incidence angles of *θ* < 50°, the bandwidth is reduced only with a couple of tens of nanometers. For *θ* < 50° incident light angle, the absorption for both TE and TM polarizations is still above 0.8 within our desired wavelength range. However, for larger angles, the high cavity absorption response is no longer sustained and the absorption capacity of the multilayer stack diminishes considerably. Although, in small angles of incidence, the overall response of TM polarized light is better compared to that of TE one, when the oblique angle gets larger the TM polarized light absorption is hampered significantly. In extremely wide angles, TM light has a very small transverse component while its perpendicular part is dominant. Therefore, due to the satisfaction of the boundary conditions on the surface of metal, the layers acts as a perfect electric conductor (PEC) in which most of the impinged light is reflected back. But this is not the case for TE polarized light where the transverse component of the E field has constant amplitude and direction.

**Figure 5:**
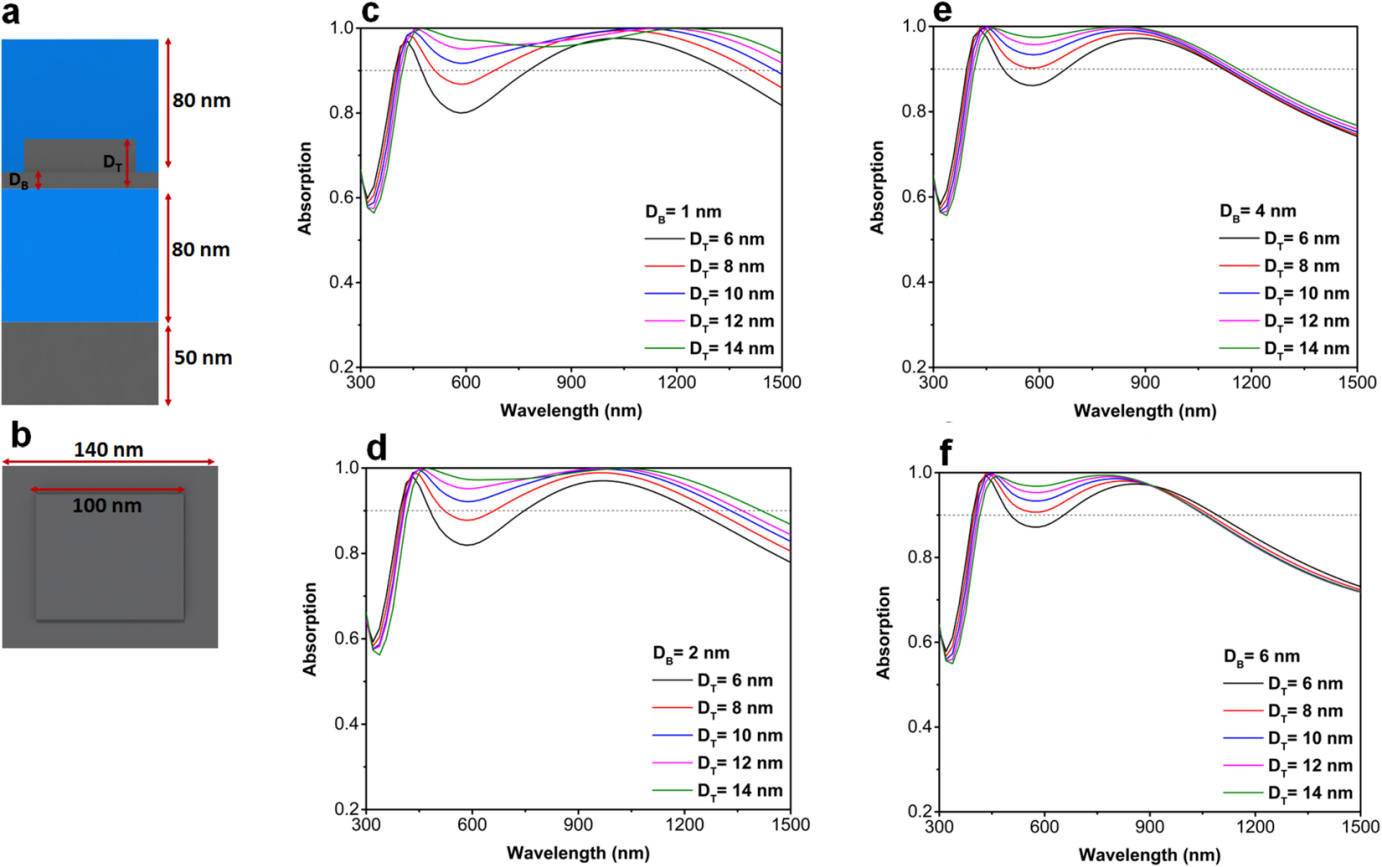
Schematic illustration of (**a**) cross sectional and (**b**) top views of the proposed unit cell of the simulation. Dependence of absorption spectra of the MIMI stack into top and bottom metal layer thicknesses for different metal bottom metal thicknesses of (**c**) ***D***_***B***_ = 1 nm, (**d**) ***D***_***B***_ = 2 nm, (**e**) ***D***_***B***_ = 4 nm, and (**f**) ***D***_***B***_ = 6 nm.

The proposed MIM multilayer is essentially a low quality-factor asymmetric Fabry-Perot design that confines light in the middle metal layer, see Fig. 3c. This planar architecture is essentially composed of an MIM cavity that has been gradually matched to the air with an antireflective coating. The overall response of this design is determined by the optical properties of each metal and insulator layers. Therefore, in the planar case, the overall bandwidth is restricted with an inherent optical characteristic of the metal and insulator coatings. Looking back to Fig. 2b, we can find that the absorption edge is longer for thinner metal layers but thicker coatings have a perfect flat response in lower λs. Therefore, an optimal design should merge the advantages of both thicknesses to be able to cover a broad frequency range. This condition substantially improves the absorption bandwidth of the planar design. In order to examine this statement, we first conducted FDTD simulations on a simplified structure. Figure 5a describes a cross sectional view of the proposed structure where the metal layer has a non-planar multi-thickness surface texture. The layer has a thin part with a thickness of D_B_ and a thick part with a thickness of D_T_. As illustrated in Fig. 5b, in the simulated unit cell the thick layer dimensions are 100 *nm* × 100 *nm* while these values for the bottom thin one are chosen to be 140 *nm* × 140 *nm*. These dimensions are chosen to provide an area filling factor of 50% for two different layers. To understand the absorption capability of this non-uniform texturing, we sweep the amount of D_T_ for fixed values of D_B_. As shown in Fig. 5c, for the case of *D*_*B*_ = 1 *nm*, the absorption of the structure is much stronger as compared to that of planar designs. This panel demonstrates that the absorption edge and strength of the MIMI structure are substantiated when we move to thicker D_T_ values, i.e. In other words, as the ratio of D_T_/D_B_ is increased the absorption bandwidth of the multilayer stack is improved. For instance, for the case of *D*_*T*_ = 10 *nm*, again keeping 0.9 as the perfect absorption threshold, the absorption edge is located at 1480 nm and this moves to larger values for thicker D_T_s. The same trend is followed for the case of *D*_*B*_ = 2 *nm*, but the overall absorption bandwidth is smaller compared to that of 1 nm thick layer (see Fig. 5d). For the D_B_ values of 4 nm and 6 nm, the thickening of the top metal (D_T_) can only improve light absorption in shorter wavelengths but does not extend the absorption edge anymore, see Fig. 5e,f. This is expected when taking the pervious results into consideration. Figure 2b proved to us that the absorption edge can only be extended if we use thin metal layers and this extension can be greater as we go to thinner coatings. These findings confirm that the use of a non-uniform multi-thick layer instead of planar morphology can provide a flat perfect light absorption in a wide frequency range.

**Figure 6:**
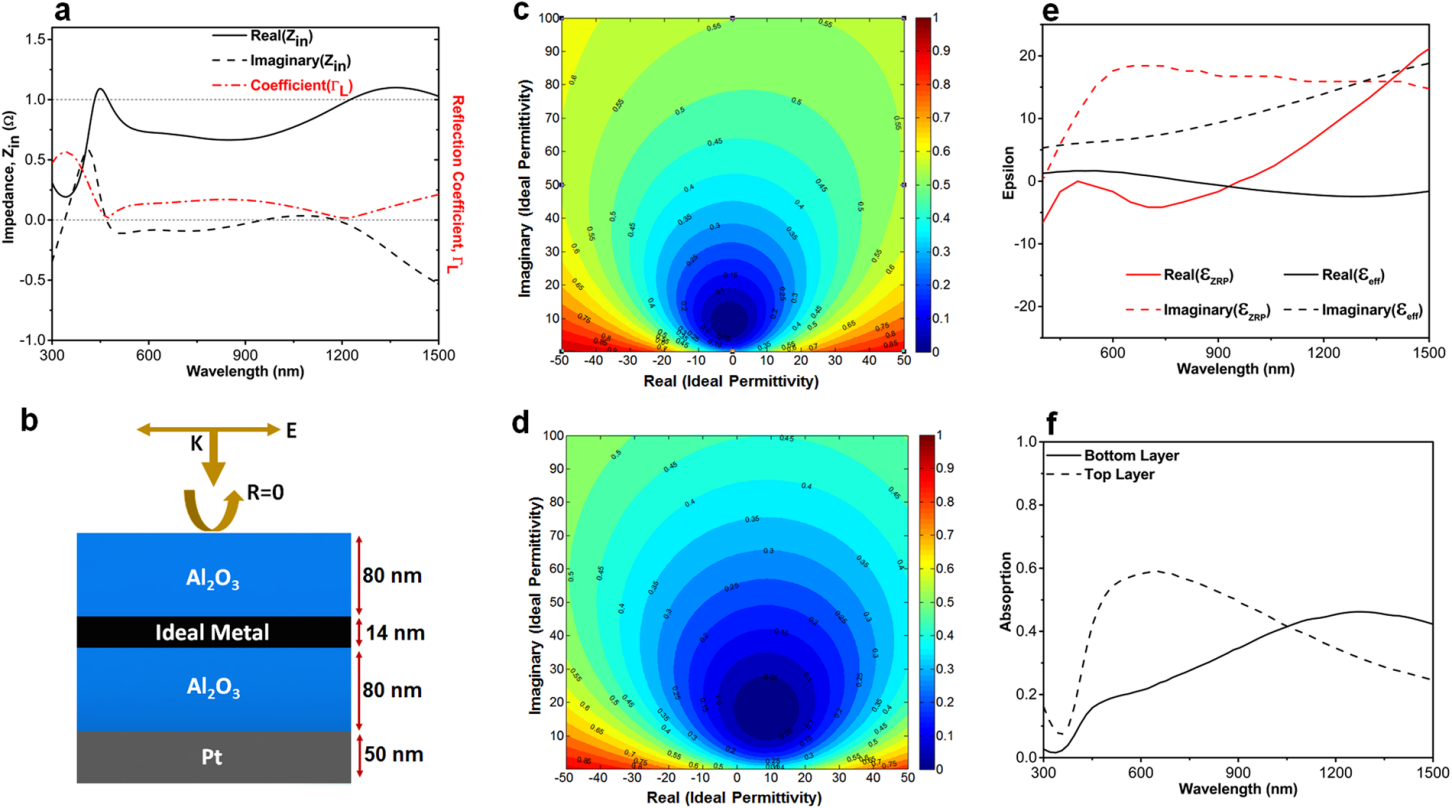
(**a**) The extracted input impedance and reflection coefficients for the non-uniform textured design with ***D***_***B***_ = 1 nm and ***D***_***T***_ = 14 nm. (**b**) Illustrative cross sectional view of the setup to find permittivity values for middle ideal metal layer and corresponding reflection contour plots for reflection values of the MIMI multilayer as a function of real and imaginary parts of permittivity at (**c**) 470 nm and (**d**) 1225 nm. (**e**) Extracted real and imaginary parts of permittivity for our nonuniform textured design and zero reflection point (ZRP) of ideal perfect absorber. Part (**f**) demonstrates the total absorption amount at the bottom (manitor A) and top (manitor B) metal layers as a function of excitation wavelength.

To get more insight into the origin of this improvement, using the aforementioned effective parameter extraction, we calculate the input impedance of the MIMI structure. Figure 6a illustrates that the real part of the input impedance retains around unity while its imaginary part is close to zero, throughout a broad wavelength range. Similar to Fig. 3a, we have also calculated the reflection coefficient amount where two dip points in reflection are recorded at 470 nm and 1220 nm. In these wavelengths, the input impedances are *Zin* = 1.01 − *i*0.01 and *Zin* = 0.98 − *i*0.03 which are very close to the perfect impedance matched condition. In the second step, we need to compare the effective permittivity value of the stack with that of an ideal metal. However, due to the non-planar nature of our design, we cannot use Eq. 1 to extract ideal permittivity values considering the fact that it is not possible to define d_Pt_ for this non-uniform design. To be able to determine ideal middle metal layer for this configuration, we used transfer matrix method (TMM). For this aim, the permittivity values of the middle metal layer is taken as an unknown complex function of frequency to satisfy the zero reflection condition for the MIMI stack. Figure 6b illustrates the dimensions that we utilized to find the ideal metal layer. Figure 6c,d depict the contour plots for the reflection of the MIMI stack as a function of real and imaginary part of ideal metal layer for the two different wavelengths of 470 nm and 1220 nm at which the reflection is zero. Zero reflection points (ZRPs) for these two wavelengths are calculated to be *ε*_*ZRP*_ = −0.83 + *i*8.42 and *ε*_*ZRP*_ = −8.84 + *i*15.92, respectively. Moreover, the *R* = 0.1 contour has smaller radius for shorter wavelength. This means that reflection enhancement due to deviation from *ε*_*ZRP*_ value is less significant for longer wavelengths. Extracting *ε*_*ZRP*_ values for different wavelengths, we have compared our effective permittivity amounts with those of the ideal case (Fig. 6e). It is obviously seen that there is a fair agreement between the imaginary parts of epsilons throughout the whole region. However, moving toward longer wavelengths, this matching is gradually lost for the real part of permittivity. In other words, the lack of matching in the real parts of ideal and extracted permittivity values is the reason that the absorption capacity of the MIMI stack lessens in longer λs. To provide a comprehensive study, we have also compared the matching condition for other noble metals such as Au and Ag which have been frequently employed in absorber designs^28–32, 42^. As it can be seen from Fig. S2, the real and imaginary parts of permittivity values for Au and Ag have very poor matching with the ideal case. Therefore, these metals cannot offer a good performance in MIMI configuration. This has been numerically examined as well as shown in Fig. S3. As this figure illustrates, Au and Ag can offer a bandwidth which is much narrower compared to that of Pt. This can be also confirmed looking at the previous studies where an absorption bandwidth narrower than 200 nm have been reported using Ag and Au nanoparticles. Therefore, to extend absorption toward longer wavelengths, Pt is much better compared to these metals. In addition to this, another reason that makes Pt an ideal choice for perfect light absorber is its durability and high structural stability. Considering the high absorption of the light (above 0.9) in an ultra-broadband regime, metal can reach to high temperatures upon exposing to the light. Therefore, it can experience corrosion, oxidation and melting which hamper its optical performance. However, Pt is a non-corrosive, non-oxidant metal which has high melting temperature compared to Au and Ag. In addition to these results, we have also calculated the amount of absorbed power in two different parts of the multi-thickness middle metal; top and bottom layers. Monitors A and B have been shown in the inset of Fig. 6f. As we can see in this figure, in the shorter wavelengths, most of the absorption is concentrated in the top layer while this dominancy is shifted into bottom layer for longer ones. This is also in line with our assumption that the absorption upper edge is defined with the thinner bottom layer.

**Figure 7:**
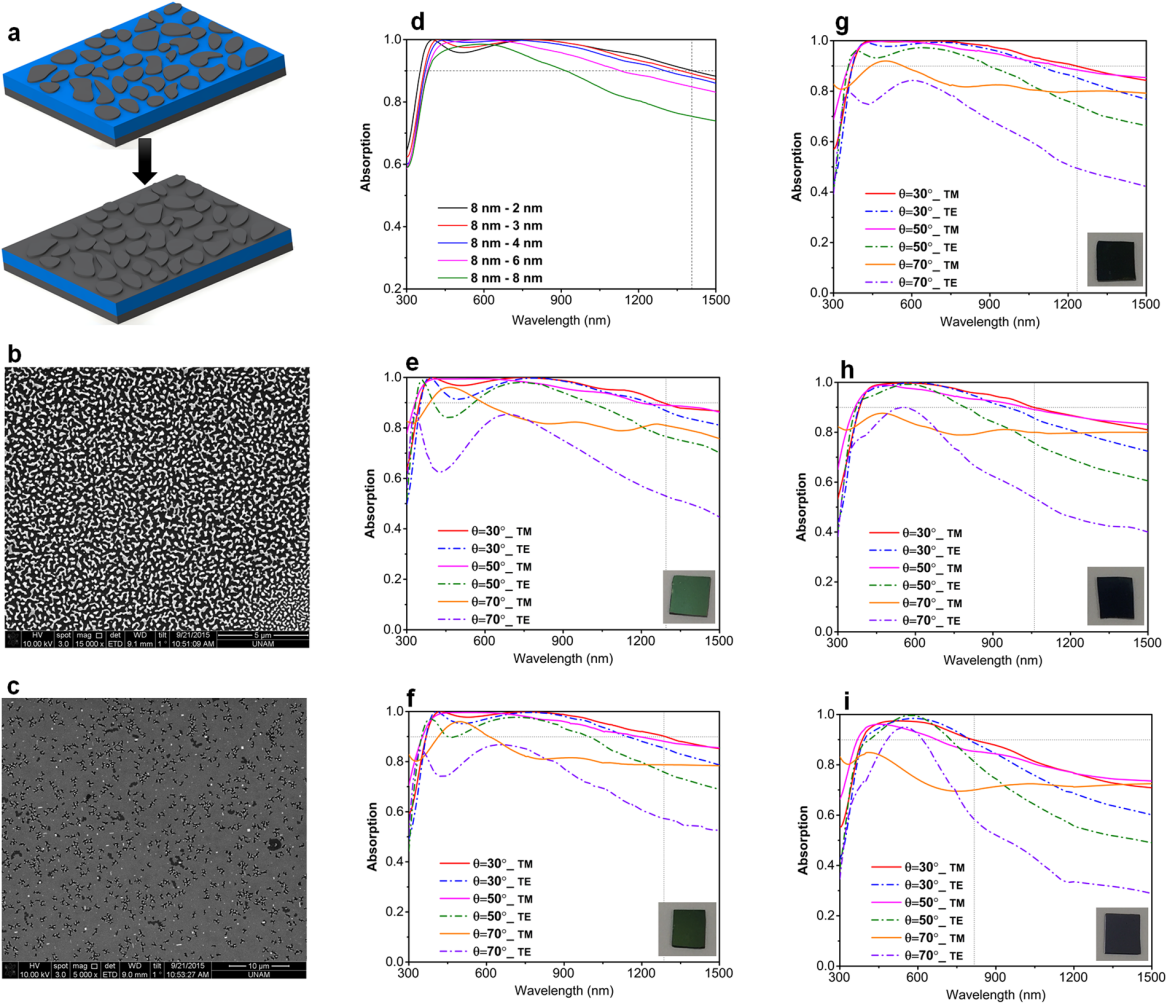
The proposed approach to obtain non-uniform textured configuration using dewetting process and SEM images of (**b**) thin (8 nm) and (**c**) thick (14 nm) Pt layer annealed at 800 °C. (**d**) Absorption spectra of fabricated samples with different configurations for normal incidence excitation. Angular dependence of absorption for different angles of incidence and polarizations (TE and TM) for the cases of (**e**) 8 nm–2 nm, (**f**) 8 nm–3 nm, (**g**) 8 nm–4 nm, (**h**) 8 nm–6 nm, and (**i**) 8 nm–8 nm. Inset shows angled views of the fabricated MIMI multilayers.

Now to fabricate the proposed design, we need to develop an EBL free, large scale compatible approach so that the design preparation simplicity stays intact. To satisfy these requirements, we utilize a simple dewetting process to obtain nanostructures from the planar film. Dewetting is a process in which a thin metal layer is annealed in a specific temperature for a desired duration. Based on the annealing temperature and duration nanoholes or nanoislands can be accomplished. When a metal layer is annealed in a temperature close to its melting point, random nanoholes start to grow throughout the layer and by passing the time these nanoholes expand and touch the neighboring ones. Consequently, nanoholes turn into nanoislands that are randomly size distributed and positioned in different separations from each other. By increasing the annealing time, these nanoislands can even get more separated. In our case, we need to have dense Pt nanoislands in which this nanoislands will be later coated with a thin Pt layer and overall a non-uniform metal layer can be achieved. To obtain this configuration, two different metal layers with thicknesses of 8 nm and 14 nm are deposited using ALD. Afterward, these layers are annealed in a tube furnace at 800 °C for an optimized duration of 8 min. Considering the high annealing temperature of the design, the morphology of the first two layers have been also investigated. Figure S4(a) illustrates the magnified cross sectional view of the multilayer design. As it can be clearly seen, all four layers can be easily distinguished without any diffusion from top or bottom Pt layer into alumina layer. Moreover, to have a better understanding about bottom Pt layer morphology, we separately deposited a 50 nm Pt layer on a quartz substrate and annealed it in the same conditions. As we can see from Fig. S4(b), the annealing has not created any pin holes (or nanoislands) in the Pt layer which is something expected considering the high layer thickness and short annealing time. Later, these metals are brought back into the ALD chamber and these nanohole/nanoisland structures are covered with a thin Pt layer. Figure 7a schematically illustrates the preparation route of the sample. SEM images in Fig. 7b,c shows the morphology of the dewetted 8 nm and 14 nm Pt layers. As we can see, the thin Pt layer is turned to densely packed nanoislands while for the thick layer, nanoholes have appeared after the annealing procedure. This is expected considering the fact that in thicker layers melting and recrystallization is much more difficult compared to that of a thin layer. By increasing the annealing time, we can also turn 14 nm thick Pt layer into nanoisland but the output is a highly separated nanoisland morphology which is not our desire. SEM image of the dewetted 14 nm thick Pt layer for a duration of 15 min is depicted in Fig. S5 (supplementary information).

To evaluate the results presented in the previous sections, using the abovementioned dewetted samples, we fabricated the non-uniform thick Pt layers based MIMI stacks. For this aim, the dewetted 8 nm Pt layer (as shown in Fig. 7b) is coated with different Pt thicknesses of 2 nm, 3 nm, 4 nm, 6 nm, and 8 nm. Considering the fact that Pt layer formation in the ALD process is based on nucleation of Pt nanoislands, 1 nm Pt layer cannot be continuous and we have not investigated this case in our fabrications. This has been proved from SEM image shown in Fig. S6(a,b) where the layer morphology for 1 nm and 2 nm thick Pt layers has been depicted. Figure 7d depicts the absorption response of different configurations over a wavelength range of 300 nm–1500 nm. It should be noted that the annotation used to describe different cases is as “the thickness of bottom dewetted metal (nm) – the thickness of coated metal layer (nm)”. Therefore, for an instance, 8 nm–2 nm means a case that 8 nm dewetted Pt layer is coated with a planar layer with a thickness of 2 nm. Therefore, in overall, for this configuration ${D}_{T}\cong 10\,nm$ and ${D}_{B}\cong 2\,nm$. As Fig. 6d shows, in the case of 8 nm–2 nm, the absorption bandwidth is the highest and the absorption edge is extended to wavelengths as large as 1410 nm. The absorption bandwidth for this case is approx. 1060 nm (350 nm–1410) that is much wider compared to that of optimized planar case which was 620 nm. The absorption edge for the cases of 8 nm–3 nm, 8 nm–4 nm stays almost unchanged with a small blue shift. However, for thicker layers, this shift becomes significant and the stack optical performance is hampered. However, thicker layers have stronger absorption in shorter wavelengths. The overall trend for different cases is in line with our simulation results depicted in Fig. 5. In general, it can be stated that as the aspect ratio between the bottom and top metal layers thicknesses (D_T_/D_B_) increases, the overall absorption bandwidth of the MIMI stack strengthens. Moreover, the angular dependence of the absorption for TE and TM polarizations has been studied in this figure as well, see Fig. 7e–i. For TM polarizations, all the configurations demonstrate an absorption that is above 0.8 throughout the entire 300 nm–1500 nm range. However, TE polarization absorption has a weaker response specifically for *θ* ≥ 50°. However, overall, these absorption spectra show that this absorber has not only an ultra-broadband response but also it retains its high absorption even in wide angels of incidence. The results obtained in this study are also extendable to other types of MI pairs based absorbers where a simple, large scale compatible annealing step can create multi-thickness patterns in the middle metal layer and this in turn would improve light absorption strength and bandwidth.

## Conclusion

In summary, in this study, we have developed a facile and large scale compatible fabrication route to grow metal-insulator multilayers for perfect absorber application. To this aim, the ALD method is utilized to grow all the layers with a high precision level. Afterward, to get a non-uniform surface texture from the planar design, the dewetting process is employed. As the consequence of dewetting process, planar layer turns to a nanoisland case. Afterward, this nanoisland morphology is uniformly coated with a second thin layer and the output will be a non-uniform multi-thick metal layer. It is shown that the existence of different metal thicknesses will ensure light absorption in a wide frequency range. It is demonstrated that the thicker part of metal gets activated in shorter wavelengths while absorption in longer wavelengths is mainly due to thinner layers. The proposed idea has been also fabricated and characterized. The absorption bandwidth for the planar architecture is 620 nm (380 nm–1000 nm) in the optimum case, while this value for the multi-thick non-uniform metal layer based MIMI is much wider with a bandwidth of 1060 nm (350 nm–1410 nm). These findings prove that a simple surface texturing, without increasing the overall thickness of the stack, can significantly boost the overall performance of the device. This approach can be applied to other types of metal-insulator multilayers based architectures (used for light manipulation and confinement applications) where an ultrathin absorber with a broad and wide angle response is of great interest.

## Electronic supplementary material

Supplementary Information
