# Supplementary material for: Ultra-broadband, wide angle absorber utilizing metal insulator multilayers stack with a multi-thickness metal surface texture
Source: Sci Rep. 2017 Jul 6;7:4755. doi: 10.1038/s41598-017-04964-3 (PMC5500529; doi:10.1038/s41598-017-04964-3)
Supplement: Supplementary file 1 — Supplementary Information [file 41598_2017_4964_MOESM1_ESM.pdf]

Supporting Information

SUBJECT AREAS: METAL-INSULATOR MULTILAYER, PERFECT ABSORBER, ULTRA-BROADBAND, ATOMIC LAYER DEPOSITION, CAVITY

Correspondence and requests for materials should be addressed to Prof. Dr. Ekmel Ozbay (E-mail: ozbay@bilkent.edu.tr)

Ultra-broadband, wide angle absorber utilizing metal insulator multilayers stack with a multi-thickness metal surface texture

Amir Ghobadi[1, 2, *], Sina Abedini Dereshgi[1, 2], Hodjat Hajian[1], Berkay Bozok[1, 2], Bayram Butun[1], Ekmel Ozbay[1, 2, 3, 4, *]

[1] NANOTAM-Nanotechnology Research Center, Bilkent University, 06800 Ankara, Turkey

[2] Department of Electrical and Electronics Engineering, Bilkent University, 06800 Ankara, Turkey

[3] Department of Physics, Bilkent University, 06800 Ankara, Turkey

[4] UNAM-Institute of Materials Science and Nanotechnology, Bilkent University, Ankara, Turkey

* Corresponding authors: amir@ee.bilkent.edu.tr and ozbay@bilkent.edu.tr

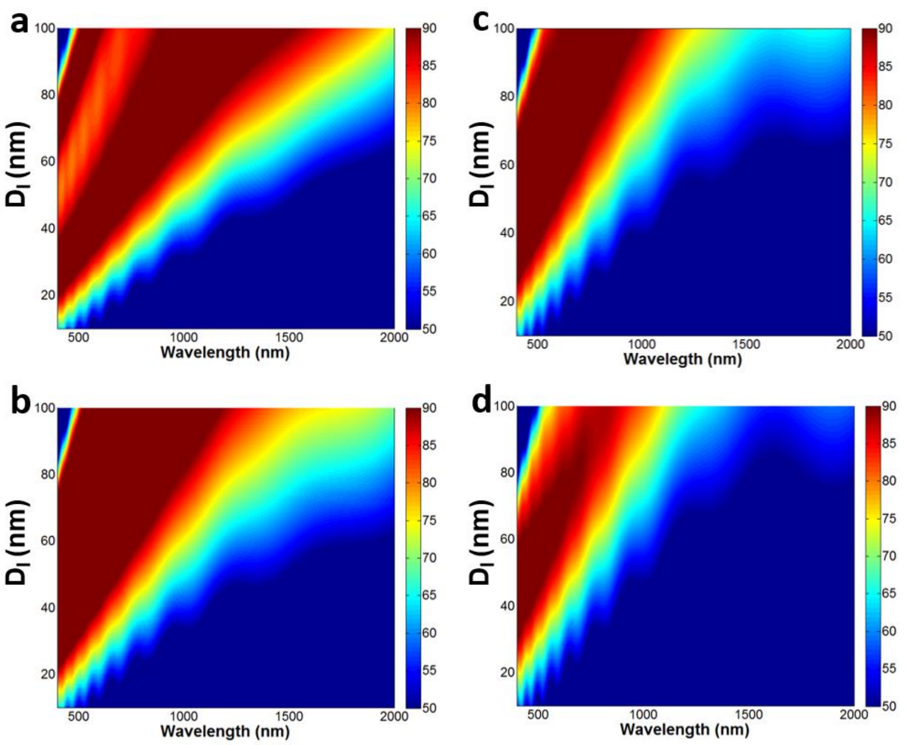

Figure S1. Absorption plot as the function of light wavelength and insulator layer thickness for different metal thicknesses of a) D_M=5 nm, b) D_M=10 nm and c) D_M=15 nm, and d) D_M=20 nm

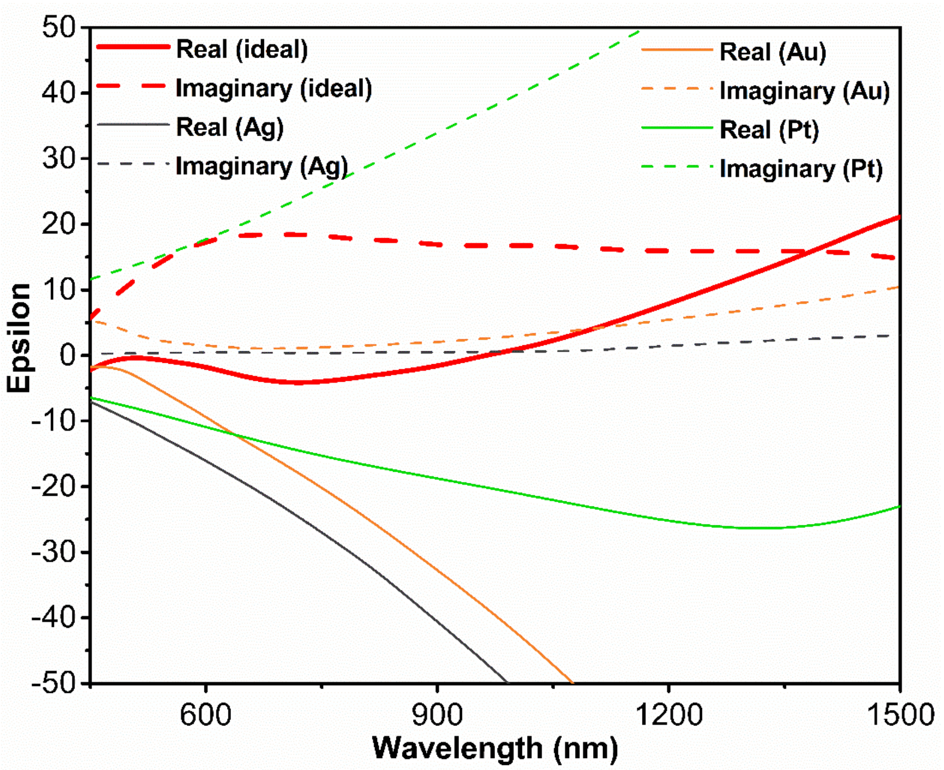

Figure S2. Permittivity values for different metals and their matching with the ideal case.

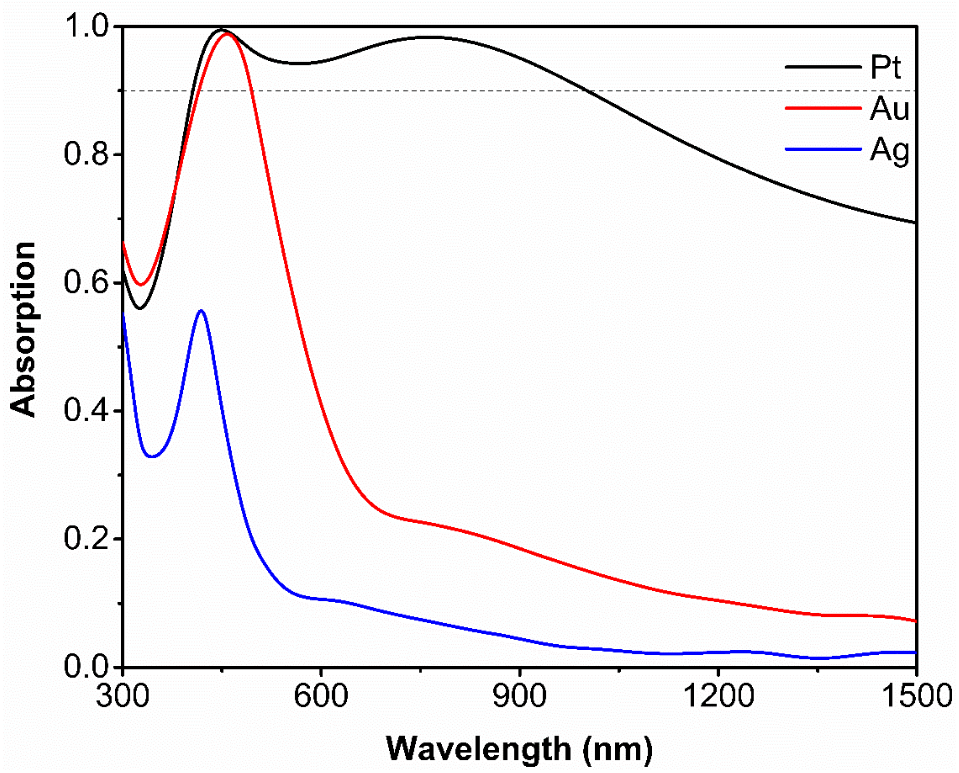

Figure S3. The absorption spectra for MIMI absorbers made of Pt, Au, and Ag metal layers.

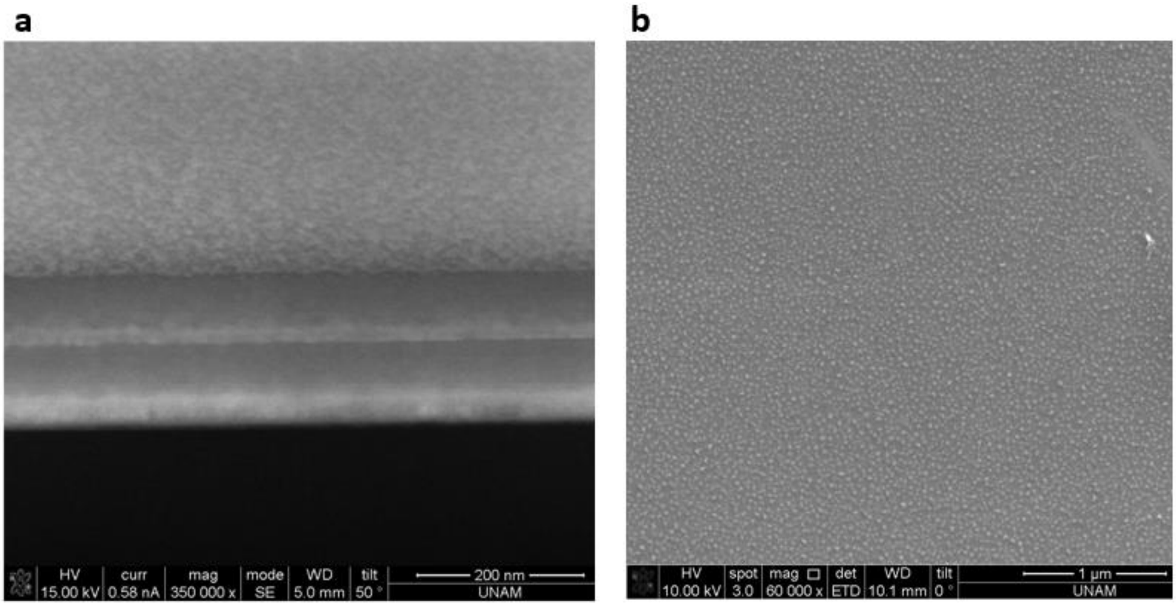

Figure S4. (a) Cross sectional and (b) top view SEM images from the MIMI multilayer structure and bottom Pt layer after annealing.

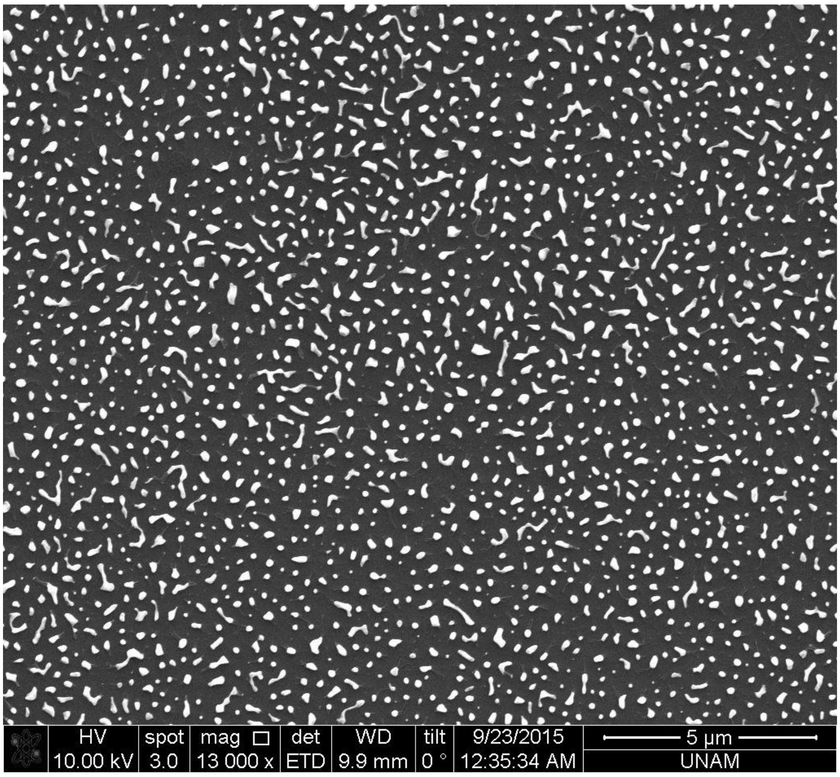

Figure S5. The SEM image of dewetted 14 nm Pt layer for a duration of 15 min.

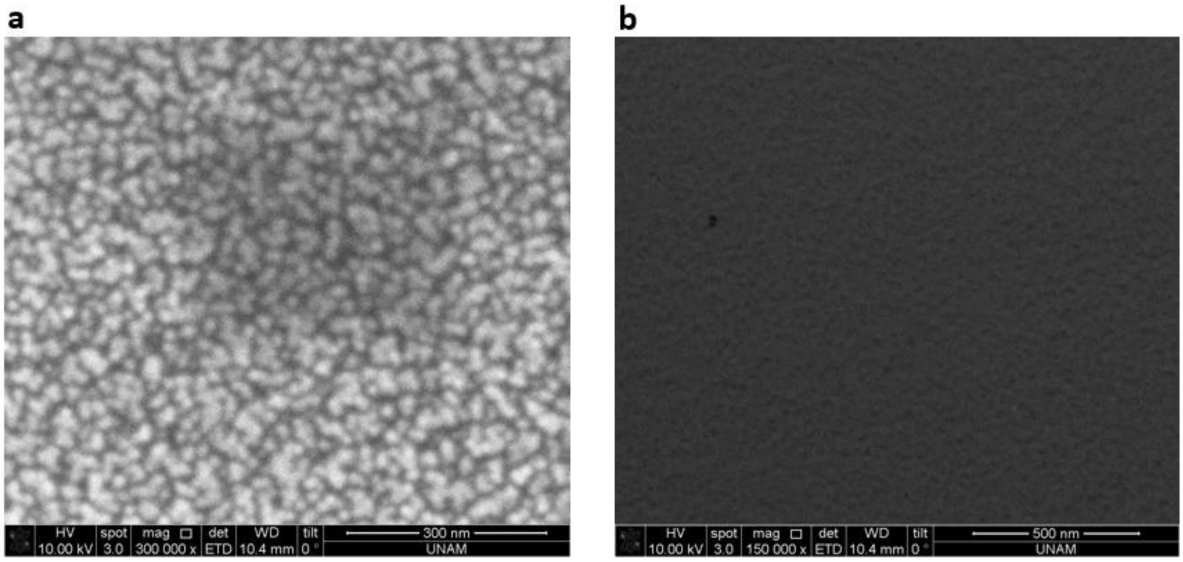

Figure S6. (a) Top view SEM images from (a) 1 nm and (b) 2 nm thick Pt layer deposited on the Alumina coated quartz substrate.
